# A new generation of physicians—The Generation Z. Are you ready to deal with it?

**DOI:** 10.3389/fpubh.2022.1015584

**Published:** 2023-01-09

**Authors:** Narcisse Elenga, Guha Krishnaswamy

**Affiliations:** ^1^Department de Pediatrie, Hôpital Andrée Rosemon, French Guiana, France; ^2^Wake Forest School of Medicine, Winston-Salem, NC, United States

**Keywords:** new generation physicians, Generation Z, flexibility of tasks, doctor's well-being, good treatment at work

What does Generation Z mean? Generation Z is the name given to the current generation of young people by many demographic researchers. According to the Pew Research Center (1), Generation Z is the generation of people born between 1997 and 2010. It is the successor to Generation Y and precedes Generation Alpha. It is defined as the first generation to be born into a world with the Internet, smart devices and apps. As a result, these individuals have radically different views on the meaning of privacy, trust and relationships in the digital world. They are also more dedicated to overall personal wellness—but everything it encompasses, including economic security, nutrition, fitness, sleep and stress management. Members of Generation Z are fighting for social change, racial equity and environmental protection. They are also more likely to be highly educated and include many participants in medical professions. These are aspects that the healthcare industry has never addressed.

Caring for patients is a form of priesthood, we've been taught, and we know stories of physicians who have dedicated their lives to caring for others, some even losing their lives in the process (for example during wars, environmental disasters and pandemics). The recent experience with the COVID-19 outbreak has shown us how physicians have risked their lives to save patients.

I will always remember my medical school days, the hospital internships during which I wished I had more and more work to learn more, and especially to learn as much as possible. I enjoyed the difficult shifts, during which we spent the whole night working non-stop. And the next day, my colleagues and I were curious to know the fate of the patients we had seen during the shift. I also know doctors who work without counting the hours, without respite, always ready to help their colleagues in difficulty, at the risk of sacrificing their family life (2). Have times changed today? Physician suicide is an emerging and very concerning issue today. The current literature announces more alarming statistics (3, 4). Indeed, extended hours of intense work with sometimes complex patients can lead to mental and physical fatigue. As a result, this may explain the risk for suicide among physicians.

Some physicians have experimented with shorter work hours in the hope of circumventing fatigue, burnout and depression. I have chosen here to describe the experience of a young colleague, 25 years old, who chose to work 5 hours a day from Monday to Friday. This colleague keeps a sports activity, 2 h a day, from Monday to Friday. He explains his choice by saying that he wants to give priority to his comfort, his moral and physical well-being, and his family life. He indicates that some physician work can be done by telecommuting. He also argues that he chose to be a doctor in order to be of service to other human beings, but he also feels strongly that taking care of himself should be a priority. He says that doctors are often insensitive to their own dire needs. They burn both ends of the candle and accumulate a rather large nest egg toward the end of their lives. They often plan to take their many remaining days off at the end of their career, but unfortunately, many will die before their retirement date or develop some incapacitating illness. Moreover, the medical profession having taken a personal toll on their personal, social lives- they end up dying alone. Their families often detest the fact that, they care more about their jobs and their own finances than their own kith and kin. These older generation physicians appear to be only thinking about their profession, to the detriment of their families and their own well-being. They are victims of the health care system that demands more and more investment, but which does little to improve the working conditions. Sadly, most health authorities do not care about the amount of work doctors do since what matters to them is sheer productivity. They do not care about the quality of life at work. On the other hand, younger generation doctors often see things in a radically different manner. Like my 25-year-old colleague, younger generations say they want to have a choice in how they live their profession; they don't want to be subjected to it. They say that work should be fulfilling, not exhausting. They have chosen the profession of physician with full knowledge of the facts. But from there to being totally over whelmed, they say no. In the next few years, we should expect a generation of doctors who will work less and less, who will be more and more demanding about the way they are treated by the health authorities and who will ask for more means to work better. Under these conditions, should we change the way we live our lives as physicians? Are the health authorities ready to mobilize more resources to recruit more doctors?

The two generations, often practicing side-by-side, need to learn to live and work together in harmony, despite different outlooks. This is also in the patient's best interest, as healthy doctors are more capable of making rational, evidence-based decisions. In addition, this new generation has radically different access to medical knowledge. Indeed, a smartphone contains the equivalent of a library of several dozen textbooks. A recent article can be accessed in real time during a medical meeting. The assistant or resident thus has direct and extraordinarily simple access to the same information as the seniors and the head of the department, which can call into question (and this is very good!) a hierarchy of knowledge, sometimes locked in a pyramidal manner, and formerly essentially based on experience and not necessarily on knowledge.

With a very peculiar (?) way of life and a paradigm-shifting attitude that challenges the “pecking order” and the existing medical hierarchy, the digital natives, as they are called, are going to disrupt the medical world (5). This evolution is forcing managers and healthcare systems to adapt. With the arrival of these young people whose ambitions are different from those of their elders, the medical world will undergo profound changes. Indeed, Generation Z advocates equality, with a more direct, more personal relationship. Listening, trust and transparency are their main expectations. If the hospital conveys a rather strict and stressful image, its previously established codes are going to be disrupted as these young doctors are no longer looking for a stable job. As a result, a diversification of tasks is going to become mandatory and considered beneficial to their blossoming. Challenges, objectives, punctual missions are going to punctuate the ideal professional daily life of this generation. Indeed, because of their close relationship with the digital world, they will be able to manage several tasks simultaneously. For these young people of Generation Z, the new digital tools need to be designed such that they do not disrupt the rhythm of work, while the management of schedules must be more flexible, and the transition from the clinic to the home be made more fluid. Management will quickly undergo radical changes in its organization. After adapting, as the healthcare system will have to do, managers will have to think about another, equally important point: what can they do to retain this generation of ultra-mobile doctors? In addition, with the feminization of the medical profession, this will change the game even more. The priority will be the family, and maybe not the hospital. It should be noted that this is also an issue for nurses and clearly explains the current shortage in many countries. Gone is the generation of the doctor who sacrifices his family life to devote himself fully to his work? In addition to telemedicine, other solutions are certainly possible in the organization of teams to combine quality of care, professional satisfaction and quality of extra-professional life: (i) the grouping of healthcare professionals in private practice allows for coordinated practice, which has the particular advantage of allowing for a more respectful organization of professionals' time; (ii)in hospitals and private practices, certain medical tasks can also be delegated to other healthcare professionals such as medical assistants or advanced practice nurses, in order to reduce the workload; (iii) at the same time, it may be wise to allow time for relaxation and recreation with loved ones.

In any case, Generation Z physicians are already here. We will have to adapt to their way of conceiving the profession, the health authorities have been warned. Will they really have a choice?

## Author contributions

All authors listed have made a substantial, direct, and intellectual contribution to the work and approved it for publication.
